# Copper Electrodeposition on a Magnesium Alloy (AZ80) with a U-Shaped Surface

**DOI:** 10.3390/ma7117366

**Published:** 2014-11-14

**Authors:** Ching An Huang, Yu Hu Yeh, Che Kuan Lin, Chen Yun Hsieh

**Affiliations:** Department of Mechanical Engineering, Chang Gung University, Taoyuan 333, Taiwan; E-Mails: d9922005@stmail.cgu.edu.tw (Y.H.Y.); cklin918@gmail.com (C.K.L.); ff127511@gmail.com (C.Y.H.)

**Keywords:** metal and alloys, thin films, corrosion, electrochemical reactions

## Abstract

Cu electrodeposition was performed on a cylindrical AZ80 substrate with a U-shaped surface. A uniform deposition of Cu was achieved on an AZ80 electrode via galvanostatic etching, followed by Cu electrodeposition in an eco-friendly alkaline Cu plating bath. Improper wetting and lower rotational speeds of the AZ80 electrode resulted in an uneven Cu deposition at the inner upper site of the U-shaped surface during the Cu electroplating process. This wetting effect could be deduced from the variation in the anodic potential during the galvanostatic etching. The corrosion resistance of the Cu-deposited AZ80 electrode can be considerably improved after Ni electroplating.

## 1. Introduction

The potential applications of engineering light materials, such as Mg, Al, and Ti alloys, has attracted much more attention in recent years, because weight reduction of electronic and automobile components is strongly needed in order to reduce energy consumption. Among these materials, components made of Mg alloys have relatively low weights. Moreover, Mg alloys have some outstanding advantages, such as high strength to weight ratio, high thermal conductivity, superior damping capacity, good electromagnetic shielding and facile recycling. Thus, Mg alloys have been widely used as construction materials in the automotive, computer, aerospace, hand tool and cell phone industries. However, Mg alloys show poor corrosion and wear resistance, which limits their widespread usage [1]. Thus, surface treatments are necessary to improve these properties in Mg alloys. Several surface treatments have been proposed to meet the requirements of corrosion protection for Mg alloys, such as metal electroplating [2,3], conversion coatings [4,5], anodising [6], micro-arc oxidation treatment [7,8], organic coatings [9] and vapour-phase processes [10]. Of these different surface treatments, electroplating is a relatively economical and convenient method. Therefore, it is worthwhile to develop a suitable protective coating method, such as Ni, Ni/Cu, and Cr/Cu coatings, for electroplating of magnesium alloys.

Mg alloys are easily corroded in water or oxidised in air [1]. Because of its low electrical conductivity and bonding ability, this corroded surface or oxide layer could impede further electroplating of the surface. Several pretreatments on the surface of the Mg alloy have been proposed for electroplating. Many reports have shown that the activation of the Mg alloy can be accomplished first by mechanical polishing, then by dipping in an alkaline solution, and finally by treatment in an acid bath containing chromic acid or hydrofluoric acid [11,12]. In our previous work, we have shown that Cu deposition could be achieved on the surface of a Mg alloy using a pretreatment of galvanostatic etching followed by electroplating in an eco-friendly Cu-alkaline plating bath [13,14]. The activation of the surface of the Mg alloy could be deduced from the variation in the anodic potential during the galvanostatic etching. We found that an activated Mg alloy surface was obtained when the anodic potential increased to a clear potential plateau during the galvanostatic etching process. After etching in the potential plateau, the oxides and the autocatalytically deposited Cu layer on top of the Mg alloy could be removed, leading to an activated surface available for further Cu electroplating [13,14,15].

In this study, a Mg alloy (AZ80) bar was shaped into a cylinder with a U-shaped surface. The AZ80 cylindrical electrode was used as the substrate for galvanostatic etching and Cu electroplating in an eco-friendly alkaline Cu plating bath. The influence of the wetting properties and the rotational speed on the coverage of the Cu deposition on a U-shaped surface is discussed. In addition, the corrosion resistance of uncoated and coated AZ80 is electrochemically evaluated.

## 2. Experimental Section

**Figure 1:**
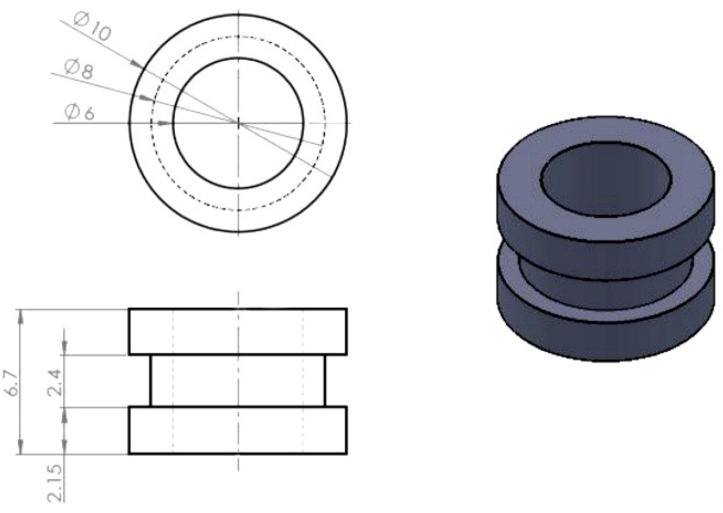
Dimensions of the AZ80 RCE with a U-shaped surface used in this study.

A bar of as-rolled magnesium alloy (AZ80) with a diameter of 13 mm was used in this study. The chemical composition of the AZ80 specimen was 8.5% Al, 0.5% Zn and 0.12% Mn with the remainder composed of Mg. Electroplating was conducted in a two-electrode cell. The AZ80 specimen was used as the electroplating substrate and shaped into a rotating cylinder electrode (RCE) with a diameter of 10 mm and a length of 6.7 mm. Figure 1 shows the dimension of AZ80 RCE with a U-shaped surface. A platinized Ti-mesh was used as the counter electrode. The AZ80 RCEs were cut into a U shape using a lathe machine to achieve a U-shaped surface for galvanostatic etching and Cu electroplating. The surface of the AZ80 RCE specimen was mechanically dry-ground with a 600-grit emery paper, ultrasonically cleaned in de-ionised water, and was subjected to galvanostatic etching followed by Cu electroplating in an alkaline Cu plating bath. Before electroplating, galvanostatic etching with an anodic current density of 20 mA·cm^−2^for 120 s was performed in an alkaline Cu sulphate plating bath to activate the surface of the AZ80 RCE. The alkaline Cu sulphate bath contained 40 g/L CuSO_4_, 150 g/L KNaC_4_H_4_O_6_, 20 g/L H_3_BO_3_ and enough NaOH to maintain pH 10.

To obtain a Cu film with a thickness of approximately 3 μm, the AZ80 cylindrical electrode was electroplated in an alkaline Cu sulphate bath with a current density of 40 mA·cm^−2^ for 600 s. After Cu electroplating in the alkaline solution, the AZ80 specimen was further electroplated in an acidic Cu plating bath with a current density of 40 mA·cm^−2^ for 410 s to effectively increase the thickness of the Cu deposition film to approximately 9 μm.

To evaluate the bonding strength between the Cu film and the AZ80 substrate, the Standard Test Method for Measuring Adhesion by Tape Test, or the ISO 2409 [16], was performed on the Cu-treated AZ80 specimens. The test was performed by scratching six parallel lines with a width of 1 mm in both longitude and latitude on top of the Cu-deposited AZ80 using a diamond knife. The scratched specimen was tightly adhered to a 3M adhesive tape (3M Company, Core Series 4-1000) for approximately 60 s, and it was subsequently peeled off rapidly in the direction parallel to the deposition surface. The bonding strength can be evaluated from the appearance of the coated specimen upon peeling. When the bonding strength is poor, the deposited film is broken and peeled from the specimen; conversely, when the bonding strength is good, only scratch marks are revealed on the surface of the specimen. According to the ISO 2409 [16], the bonding strength could be categorised into six grades from 0 to 5 in which a lower grade number corresponds to a higher bonding strength between the deposit and the substrate.

The corrosion resistance of uncoated and coated AZ80 specimens was examined by anodic polarisation measurements in a 0.1 M H_2_SO_4_ solution. The anodic polarisation test was conducted in a typical electrochemical three-electrode cell. The uncoated or coated AZ80 specimen was used as the working electrode. A platinized Ti-mesh and an Ag/AgCl electrode in a saturated KCl solution were used as the counter and reference electrodes, respectively. The anodic polarisation behaviour of the uncoated and coated AZ80 specimens was evaluated by potentiodynamic scanning with a scan rate of 5 mV·s^−1^ from −0.25 V (*vs.* open circuit potential) to the noble potential until the breakdown of the coating. The corrosion potential and corrosion current density of an uncoated or coated AZ80 specimen were estimated from its anodic polarization. It can be expected that the specimen with a lower corrosion current density could have a higher corrosion resistance.

Morphologies of the coated AZ80 were examined with an optical microscope (OM, Olympus BH2-UMA, Olympus Ltd., Tokyo, Japan) and a scanning electron microscope (SEM, HITACH S-3000N, Hitach Ltd., Tokyo, Japan) equipped with an energy-dispersive X-ray spectrometer (EDS), which allows chemical composition analysis.

## 3. Results and Discussion

### 3.1. Effect of the Rotation Speed of AZ80 RCE

**Figure 2:**
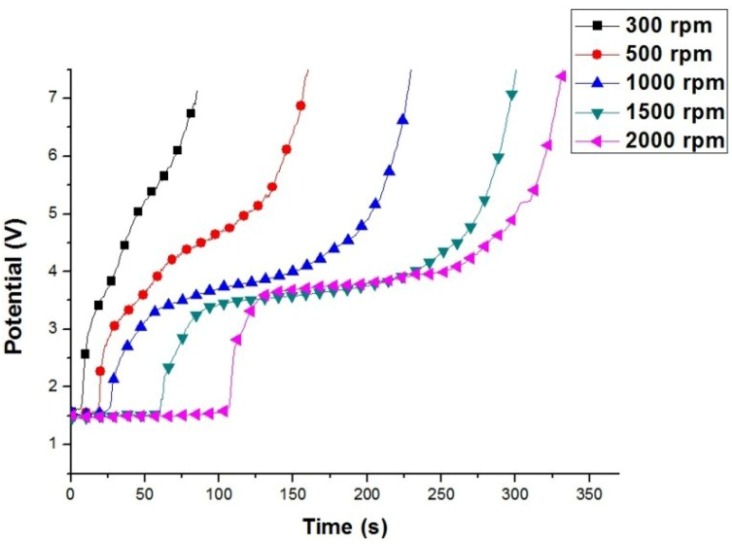
Potential variation of the AZ80 RCE at different rotation speeds during the galvanostatic etching at the current density of 20 mA·cm^−2^ in an alkaline Cu plating bath.

To study the effect of the rotation speed on the surface activation process, the AZ80 RCE was galvanostatically etched at a rotation speed that was varied from 300 to 2000 rpm. Figure 2 shows the anodic potential variation of the AZ80 RCE at different rotating speeds during galvanostatic etching in the alkaline Cu plating bath. A distinct potential plateau was clearly found in the potential variation curve when the rotation speed was 1000 rpm or greater. As shown in Figure 2, the galvanostatic etching period, marked from the beginning to the potential plateau, increased with increasing rotation speeds. At a rotation speed of below 500 rpm, the anodic potential increased steadily in a relatively shorter time, and the potential plateau was not clearly observed. In our previous study [13], we found that the activation of the surface of a Mg alloy specimen could be achieved when the anodic potential increased to the potential plateau during galvanostatic etching. With further galvanostatic etching from the potential plateau, an obvious increase in anodic potential could be seen. The potential increase is attributed to the formation of a dense oxide on Mg alloy specimen. This indicates that the Cu electroplating on the AZ80 RCE must be conducted at a rotational speed of 1000 rpm or more. The activated surface could be further treated with Cu in the alkaline Cu plating bath. This finding indicates that in order to obtain a well-adhering Cu film on the AZ80 RCE, the rotation speed of the AZ80 RCE must be 1000 rpm or greater during the galvanostatic etching and Cu electroplating in the alkaline Cu plating bath.

**Figure 3:**
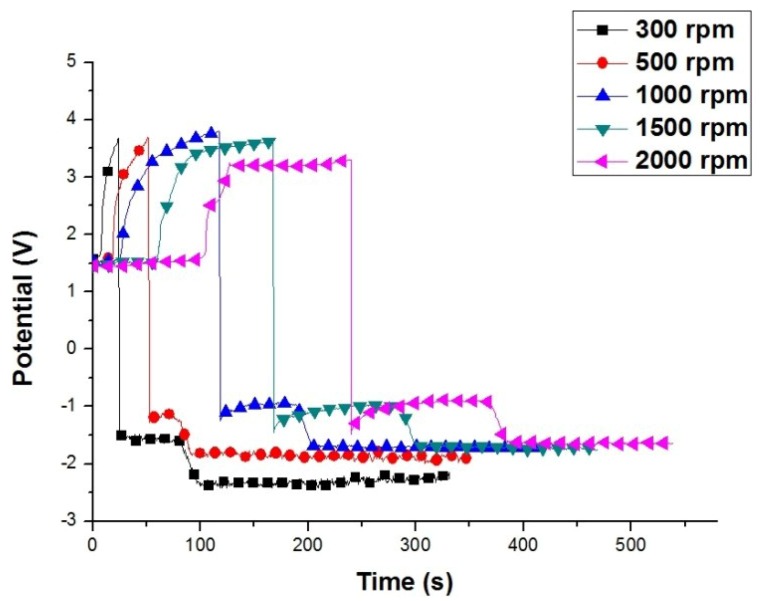
Potential variation of the AZ80 RCE at different rotation speeds during galvanostatic etching at the current density of 20 mA·cm^−2^ followed by Cu electrodeposition at the current density of 40 mA·cm^−2^ in an alkaline Cu plating bath.

Figure 3 demonstrates the potential variation of the AZ80 RCE at different rotation speeds during galvanostatic etching followed by Cu electroplating in the alkaline Cu plating bath. Although a potential plateau was not observed, the galvanostatic etching of the AZ80 RCE at a rotation speed of 500 rpm was conducted at a potential roughly corresponding to the potential plateau observed at a rotation speed of above 1000 rpm. As shown in Figure 3, the Cu electroplating at 300 rpm has an obvious potential offset of *ca.* −0.6 V. This indicates that an oxide would exist on the AZ80 RCE, leading to a little increase in cathodic potential. To evaluate the bonding strength between the Cu deposit and the AZ80 RCE, Cu electrodeposition with a plating current density of 40 mA·cm^−2^ was performed for 600 s after galvanostatic etching. Therefore, the Cu-deposited AZ80 RCEs were prepared at different rotation speeds for the bonding strength test.

**Figure 4:**
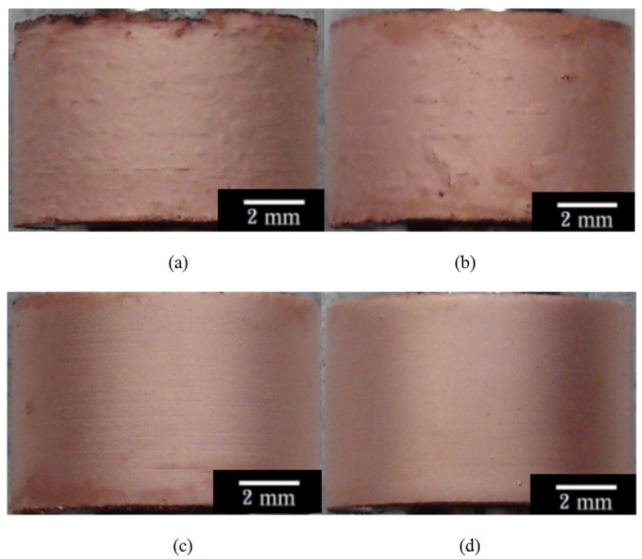
Surface morphology of the AZ80 RCE after galvanostatic etching at the current density of 20 mA·cm^−2^ and Cu-electroplating at the current density of 40 mA·cm^−2^ at rotation speeds of (**a**) 300; (**b**) 500; (**c**) 1000; and (**d**) 2000 rpm.

Figure 4a–d shows the morphologies of the Cu-deposited AZ80 RCEs prepared at rotation speeds of 300, 500, 1000 and 2000 rpm, respectively. As shown in Figure 4a,b, some blisters were observed on the Cu-deposited AZ80 RCEs prepared at rotation speeds of 300 and 500 rpm, while a uniform Cu film was observed on the AZ80 RCE at a rotation speed of 1000 rpm or greater (see Figure 4c,d). This result indicates that the bonding strength between the Cu deposition film and the AZ80 RCE could be increased significantly by increasing the rotation speed from 500 to 1000 rpm.

**Figure 5:**
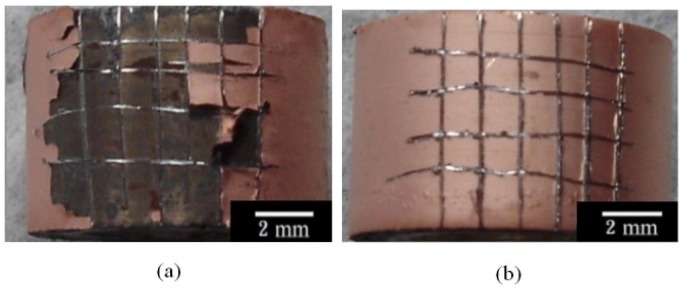
Surface morphology of the AZ80 RCE galvanostatically etched at the current density of 20 mA·cm^−2^ and Cu-electroplated at the current density of 40 mA·cm^−2^ at rotation speeds of (**a**) 500, and (**b**) 1000 rpm after the bonding strength test.

Figure 5a,b shows the surface morphologies of the Cu-treated AZ80 RCEs, which were galvanostatically etched and Cu-treated at rotation speeds of 500 and 1000 rpm in the alkaline Cu plating bath as described in the bonding strength test, known as the ISO 2409 [16]. As expected, a poor adhesion strength with an ISO grade of 5 was observed between the Cu deposit and the AZ80 substrate for the Cu-deposited AZ80 RCE electroplated at 500 rpm (see Figure 5a). In contrast, an obviously high bonding strength with an ISO grade of 0 was detected for the sample prepared at 1000 rpm (see Figure 5b). These results of the bonding strength test are in full agreement with the galvanostatic etching observations discussed earlier. That is, a potential plateau was not clearly found during galvanostatic etching when the rotation speed of the AZ80 RCE was at 500 rpm, while the potential plateau was clearly observed at 1000 rpm. An activated surface for Cu electrodeposition could be obtained when the rotation speed of the AZ80 RCE was 1000 rpm or greater. To study the Cu electrodeposition behaviour, the AZ80 RCE with a U-shaped surface was galvanostatically etched and was electroplated with Cu at a rotation speed of 1000 rpm in the alkaline Cu plating bath.

### 3.2. Effect of Wetting on the Coverage of Cu Deposition Film

**Figure 6:**
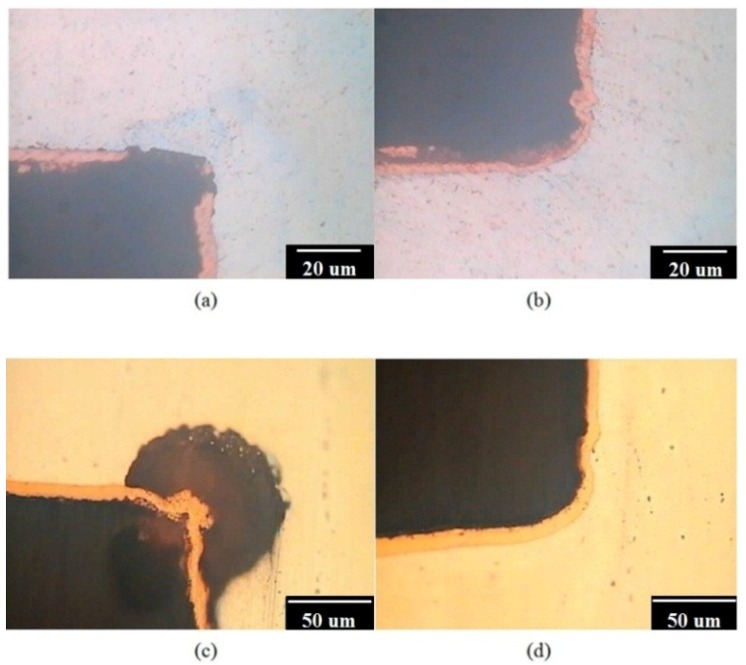
Cross sections of the inner (**a**) upper and (**b**) bottom sites of U-shaped surface of AZ80 specimen after galvanostatic etching followed by Cu electroplating in the alkaline Cu-plating bath and (**c**) and (**d**) the corresponding sites for the specimen after electroplating in an acidic Cu plating bath.

The AZ80 RCE specimen with the U-shaped surface was vertically immersed in the alkaline Cu plating solution for galvanostatic etching and subsequent Cu plating. After galvanostatic etching to the potential plateau, the AZ80 specimen was electroplated in an alkaline Cu plating bath with a current density of 40 mA·cm^−2^ for 600 s and, subsequently, in the acidic Cu plating bath with a current density of 40 mA·cm^−2^ for 410 s. From the cross-sectional micrographs shown in Figure 6a, the Cu film does not evenly cover the inner upper side of the U-shaped surface after Cu electroplating in the alkaline Cu plating bath. In contrast, a uniform and fully covered Cu deposition film is observed on the AZ80 substrate in the inner bottom side of the U-shaped surface as shown in Figure 6b. The inner upper site of U shape, where was not well covered with Cu, was not etched beforehand during galvanostatic etching and electroplating in the alkaline Cu plating bath. This finding indicates that a low corrosion rate for the AZ80 specimen can be expected in the alkaline Cu plating bath. Figure 6c,d show the cross-sections of the inner upper and bottom sites in the U-shaped surface of the Cu-deposited AZ80 specimen after Cu electroplating in the acidic Cu plating bath. The thickness of the Cu film increased markedly from 3 to approximately 9 μm after electroplating in the acidic Cu plating bath. As shown in Figure 6c, the AZ80 specimen was obviously etched in the inner upper side of the U-shaped surface after Cu electrodeposition. In contrast, an evenly covered Cu deposit was observed at the inner bottom site of the U-shaped surface. Because the AZ80 specimen was electroplated in an alkaline and, subsequently, an acidic Cu plating bath, clear etching of the inner upper site of the U-shaped surface must have taken place during acidic Cu electroplating such that the Cu film on the AZ80 specimen could show an efficient increase in thickness. Although the AZ80 specimen was rotated at 1000 rpm, it is possible that some small gas bubbles were not excluded in the inner upper corner of the U-shaped surface during immersion and etching. Therefore, the surface activation of the inner upper U-shaped site was not achieved by using galvanostatic etching. That is, the AZ80 RCE with a U-shaped surface was not properly wetted because the specimen was vertically immersed during galvanostatic etching. To enhance the wetting properties of the AZ80 RCE with a U-shaped surface, the AZ80 specimen was tilted and rotated three times to properly wet the inner upper side of the U-shaped surface before galvanostatic etching.

**Figure 7:**
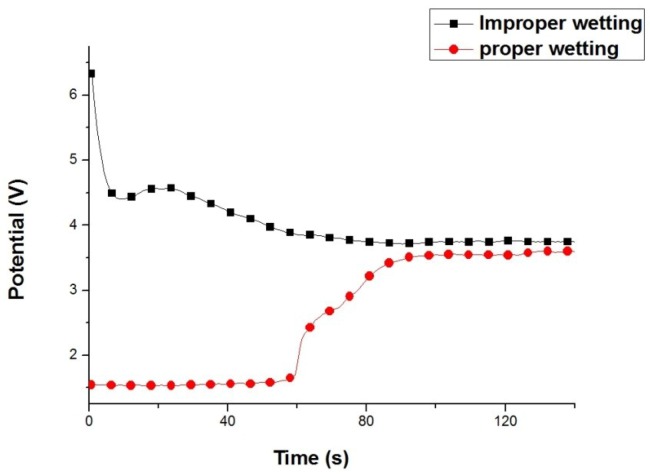
Potential variation of improperly and properly wetted AZ80 specimens with a U-shaped surface at a rotation speed of 1000 rpm during galvanostatic etching at the current density of 20 mA·cm^−2^ in an alkaline Cu-plating bath.

Figure 7 shows the potential variation of properly and improperly wetted AZ80 RCEs with a U-shaped surface during galvanostatic etching for 120 s. For the improperly wetted specimen, the anodic potential decreased rapidly from 6.4 to 4.4 V in the first 10 s and gradually decreased to 3.8 V in the subsequent galvanostatic etching. The potential plateau was not observed from the anodic potential variation during galvanostatic etching although the rotation speed of AZ80 specimen was maintained at 1000 rpm. This result indicates that the surface activation was not sufficient because the potential did not reach the lower regime for effective etching. The high potential in the beginning of galvanostic etching could be attributed to the improperly wetted AZ80 specimen. This would result from the presence of few bubbles with high impedances in the inner corners. In contrast, for a properly wetted AZ80 specimen, as shown in Figure 7, the anodic potential was steady at 1.55 V in the first 60 s and increased almost linearly to 3.75 V when galvanostatic etching was performed from 60 to 90 s and finally remained at 3.75 V, the potential plateau, until 180 s. The potential variation between the improperly and properly wetted AZ80 specimens is notably different. This finding means that the effect of galvanostatic etching on the AZ80 surface can be controlled by the degree of wetting. Moreover, the wetting effect (improper or proper wetting) could be observed from the potential variation during galvanostatic etching as shown in Figure 7.

**Figure 8:**
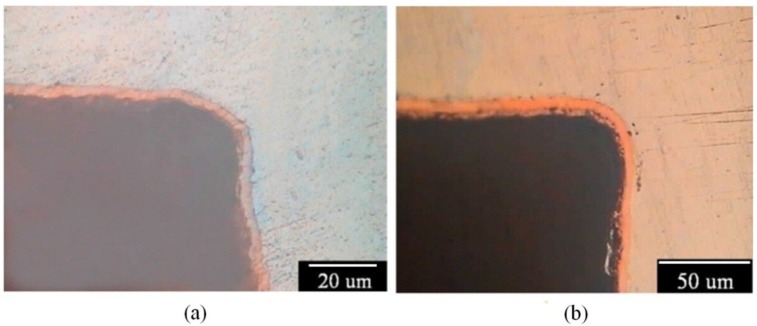
Cross sections of the inner upper sites of the U-shape surface of AZ80 specimens after proper wetting and galvanostatic etching followed by Cu electroplating in (**a**) an alkaline and, subsequently; (**b**) an acidic Cu plating bath.

**Figure 9:**
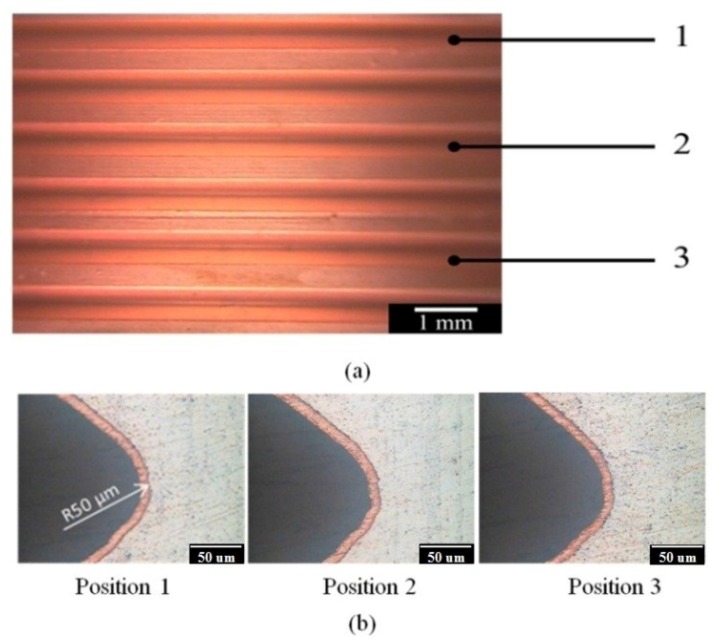
Optical micrographs of (**a**) overview and (**b**) cross section of the Cu-deposited AZ80 specimen with a multi-V shape.

Figure 8a,b shows the cross-sections of the inner upper sites in the U-shaped surface that was properly wetted, galvanostatically etched, and electroplated in an alkaline and, subsequently, an acidic Cu plating bath. As expected, a uniform and evenly covered Cu deposition film was observed in the inner upper sites of the U-shaped surface. This result means that proper wetting is needed for Cu electrodeposition on the AZ80 RCE with a U-shaped surface. An increase in the anodic potential to a potential plateau could be regarded as a suitable galvanostatic etching environment for obtaining an activated surface for further Cu electrodeposition. With proper wetting and galvanostatic etching, the AZ80 RCE with a multi-V shaped surface that resembles a screw shape could be uniformly electroplated as shown in Figure 9a,b. This finding indicates that Cu electroplating on Mg alloy components with U and V shapes is feasible. Because the AZ80 RCE with a U- or V-shaped surface could be activated and electroplated in the alkaline Cu plating bath, a Cu-treated AZ80 specimen could be the substrate for further Ni electroplating to obtain a protective Ni/Cu coating.

### 3.3. Electrochemical Test for Corrosion

**Figure 10:**
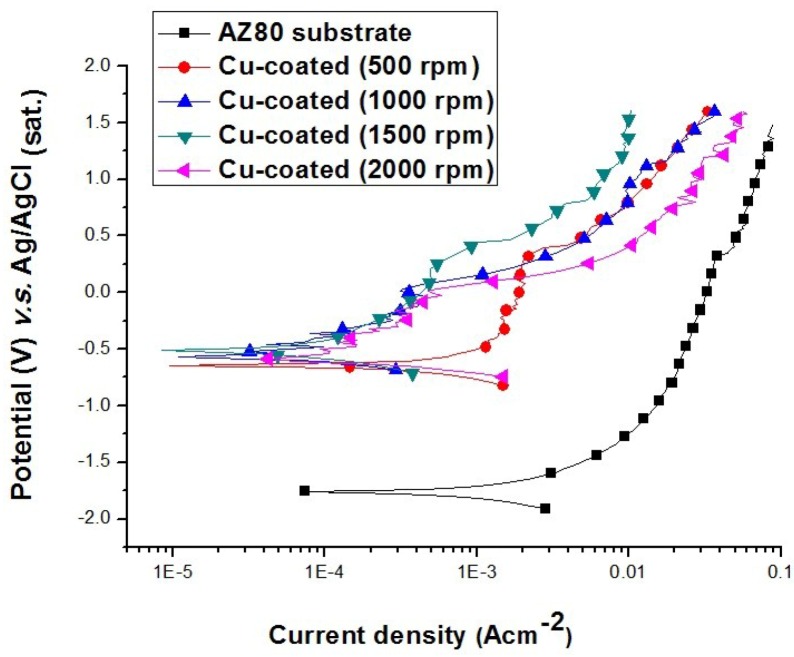
Anodic polarisation curves of uncoated and Cu-coated AZ80 RCEs with a U-shaped surface galvanostatically etched and Cu-electroplated at different rotation speeds in the alkaline bath.

**Figure 11:**
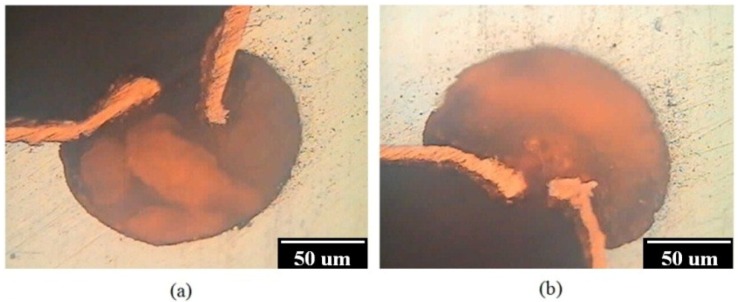
Cross sections of the inner (**a**) lower and (**b**) upper sites of U-shape surface of AZ 80 specimen prepared at a rotation speed of 500 rpm after Cu electrodeposition in the acidic Cu-plating bath.

Figure 10 shows the anodic polarisation behaviour of uncoated and Cu-coated AZ80 specimens, which were galvanostatically etched and then electroplated in the alkaline and acidic Cu plating baths at different rotational speeds in a 0.1 M H_2_SO_4_ solution. The corrosion potential of the AZ80 RCE with a U-shaped surface was −1.74 V (*vs.* Ag/AgCl), and its corrosion current density was 2 × 10^−3^ A·cm^−2^. The corrosion potential increased markedly to approximately −0.45 V (*vs.* Ag/AgCl), and the corrosion current density of the AZ80 specimen was reduced to approximately 1 × 10^−4^ A·cm^−2^ after Cu electroplating. It must be noted that the anodic current density of the Cu-treated AZ80 specimen prepared at 500 rpm is much higher than that of the specimen prepared at a rotation speed of 1000 rpm or greater. This result means that the corrosion resistance of the former is worse than that of the latter. This finding is also in agreement with the results from the bonding strength test and galvanostatic etching in which a well-adhered Cu film on the AZ80 RCE with a U-shaped surface can only be obtained when the rotation speed is 1000 rpm or greater. To examine the bonding between the Cu deposit and the AZ80 specimen prepared at 500 rpm, the cross section of the Cu-treated U-shaped surface was examined. As shown in Figure 11a,b, the inner lower and upper sites of U-shaped were strongly etched after Cu electroplating in the acidic Cu-plating bath. This result means that the Cu-coated AZ80 specimen prepared at 500 rpm has a lower corrosion resistance. This finding explains why the anodic current density was much greater than that of the Cu-coated AZ80 specimen prepared at 1000 rpm or greater.

**Figure 12:**
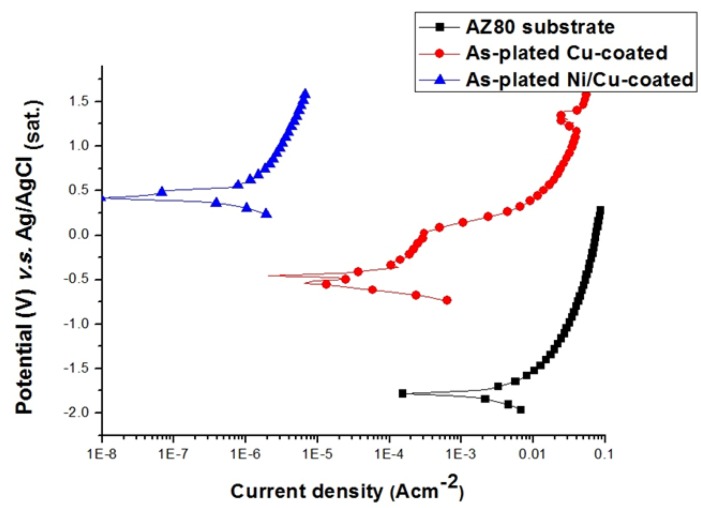
Anodic polarisation curves of Ni/Cu-, Cu-coated and uncoated AZ80 specimens with a U-shaped surface at a rotation speed of 1000 rpm.

Figure 12 shows the anodic polarisation curves of the AZ80 specimen and the Cu-coated and Ni/Cu-coated AZ80 specimens in the 0.1 M H_2_SO_4_ solution. The corrosion potential of the AZ80 specimen increased to 0.4 V (*vs.* Ag/AgCl), and its corrosion current density decreased to 5 × 10^−7^ A·cm^−2^ upon Cu and Ni electroplating. The corrosion resistance of the Cu-treated AZ80 specimen is significantly improved after Ni electroplating. That is, the AZ80 specimen with a U-shaped surface could be Cu- and Ni-electroplated to increase its corrosion resistance.

## 4. Conclusions

Cu electrodeposition was performed on a magnesium alloy (AZ80) of a cylindrical shape with or without a U-shaped surface. Experimental results demonstrated that a uniform Cu film could be produced on the AZ80 specimen through galvanostatic etching followed by Cu electroplating in an alkaline Cu plating bath. An uncovered Cu deposit was observed at the inner upper site of the U-shaped surface of AZ80 electrode when the specimen was improperly wetted and rotated at a speed of 500 rpm or lower. The wetting effect on the U-shape surface of AZ80 electrode could be detected from the variation in anodic potential during galvanostatic etching in the alkaline Cu plating bath. The corrosion resistance of Cu-coated AZ80 can be significantly improved upon Ni electroplating.
